# Synergistic and Selective Antiproliferative Effects of Cafestol and a Hyaluronic Acid–Epigallocatechin Gallate Conjugate in Human Renal Cancer Cells

**DOI:** 10.3390/ijms27114929

**Published:** 2026-05-29

**Authors:** Nunnarpas Yongvongsoontorn, Yudo Sawa, Atsushi Yamashita, Joo Eun Chung, Kaoru Hiratsuka, Koji Izumi, Hiroaki Iwamoto, Motoichi Kurisawa

**Affiliations:** 1 Graduate School of Advanced Science and Technology, Japan Advanced Institute of Science and Technology, 1-1 Asahidai, Nomi 923-1292, Ishikawa, Japan; nyong@jaist.ac.jp (N.Y.);; 2 Integrative Cancer Therapy and Urology, Graduate School of Medical Sciences, Kanazawa University, 13-1 Takara-machi, Kanazawa 920-8640, Ishikawa, Japan

**Keywords:** hyaluronic acid, epigallocatechin gallate (EGCG), cafestol, renal cell carcinoma, polymer conjugate, synergistic effects, combination index (CI)

## Abstract

Renal cancer remains a major global health burden, and current targeted and immunotherapeutic strategies are frequently limited by toxicity, therapeutic resistance, and suboptimal response rates. Natural bioactive compounds such as epigallocatechin gallate (EGCG) and cafestol exhibit anticancer activity; however, their therapeutic utility is constrained by limited potency and dose-dependent adverse effects. In this study, the antiproliferative and synergistic effects of cafestol and a hyaluronic acid (HA)–EGCG conjugate were investigated in renal cancer cells. HA conjugation significantly enhanced the antiproliferative efficacy of EGCG in both ACHN and A498 human renal cancer cells, whereas unmodified HA exhibited no intrinsic anticancer activity. Importantly, the HA–EGCG conjugate enabled a pronounced synergistic interaction with cafestol, particularly in ACHN cells, as confirmed by combination index analysis, while free EGCG and cafestol failed to achieve synergistic inhibition. In addition, the HA–EGCG conjugate and its combination with cafestol exhibited favorable selectivity toward renal cancer cells compared with normal renal proximal tubule epithelial cells (RPTECs). This combination robustly enhanced apoptosis and was associated with significant downregulation of the anti-apoptotic proteins Bcl-2 and Bcl-xL at the transcriptional level, together with suppression of the epithelial–mesenchymal transition-associated transcription factor SNAIL at both transcriptional and protein levels. Notably, the enhanced antiproliferative effects were achieved at reduced concentrations, highlighting the potential to mitigate dose-related toxicity. Collectively, these findings support HA-based conjugation as an effective strategy to potentiate the anticancer activity of natural bioactive compounds and to enable synergistic, multi-targeted therapeutic effects against renal cancer.

## 1. Introduction

Renal cancer constitutes a significant and growing global health burden, accounting for approximately 4.4% of all cancers and contributing to more than 430,000 new cases and ~175,000 deaths annually, with both incidence and mortality continuing to rise worldwide [1,2]. Although targeted therapies and immune checkpoint blockade have reshaped the therapeutic landscape, current frontline regimens, primarily tyrosine kinase inhibitors (TKIs) alone or in combination with immune checkpoint inhibitors (ICIs), remain constrained by substantial toxicity; approximately 65% of patients experience grade 3 or higher adverse events that necessitate dose modification or treatment interruption [3,4,5]. Moreover, treatment efficacy remains suboptimal: even with contemporary first-line TKI–ICI combinations, only ~55–60% of patients achieve objective responses, leaving ~40–45% with stable or progressive disease that reflects therapeutic resistance [3,4,5].

Such resistance arises from multiple, interdependent biological processes that collectively enable tumor survival and adaptation. Dysregulation of apoptotic machinery, including increased expression of anti-apoptotic Bcl-2 family proteins, diminishes programmed cell death and supports persistent tumor viability [6,7]. Additionally, activation of epithelial–mesenchymal transition (EMT) programs, driven by transcription factors such as SNAIL, SLUG, and TWIST, enhances cellular plasticity, invasion, metastatic potential, and broad drug tolerance [8,9]. Given the multifactorial nature of resistance, effective therapeutic strategies must be able to address multiple oncogenic and survival pathways simultaneously.

In this context, natural bioactive compounds have garnered increasing interest as adjunct or alternative therapeutic candidates for renal cancer due to their multi-target actions and generally favorable safety profiles. Among them, the coffee diterpene cafestol and the green tea polyphenol epigallocatechin gallate (EGCG) have demonstrated broad anticancer potential [10,11]. However, despite their activity, both compounds typically require relatively high concentrations to achieve meaningful antitumor effects, and such doses may be associated with adverse outcomes, such as hepatotoxicity in the case of EGCG or elevations in low-density cholesterol with cafestol, thereby limiting their standalone therapeutic applicability [12,13].

To address these limitations, polymer-based conjugation strategies have emerged as effective approaches to enhance molecular stability, cellular uptake, and therapeutic potency. Hyaluronic acid (HA), a biocompatible polysaccharide that interacts with receptors such as CD44, which is frequently expressed in renal cancer cells [14,15], has shown promise as a functional carrier for anticancer agents [16,17]. HA-based conjugation has been reported to improve the intracellular accumulation and pharmacological performance of conjugated therapeutics, potentially through receptor-mediated interactions and prolonged cellular exposure [18]. Consistent with this concept, our previous studies demonstrated that HA–EGCG conjugates exhibit enhanced antiproliferative activity and improved safety profiles compared with free EGCG, particularly when combined with other anticancer agents [19,20,21].

Given the complementary anticancer mechanisms of cafestol and EGCG, together with the enhanced efficacy conferred by HA conjugation, combining these interventions represents a rational therapeutic strategy. This study, therefore, investigates the synergistic anticancer potential of cafestol and the HA–EGCG conjugate, focusing on improved proliferation inhibition and the cooperative modulation of apoptosis and EMT/SNAIL-associated pathways. To evaluate potential cell line-dependent responses, experiments were performed using both ACHN and A498 renal cancer cells, which represent distinct renal cancer cell models with different biological characteristics. In addition, normal renal proximal tubule epithelial cells (RPTECs) were included to assess cytotoxicity toward non-malignant renal cells and to evaluate cancer cell selectivity. By enabling effective anticancer activity at reduced concentrations, such synergistic approaches may provide a foundation for safer and more effective natural compound-based therapeutic strategies for renal cancer.

## 2. Results and Discussion

### 2.1. Suppression of Renal Cancer Cell Proliferation

The natural compounds EGCG and cafestol possess anticancer activity; however, their therapeutic utility is often limited by dose-dependent adverse effects [13,22]. We have previously reported that HA–EGCG conjugates, in which EGCG was covalently linked to HA, exhibit enhanced antiproliferative activity and improved safety profiles when used in combination with anticancer agents [19,20,21]. In the present study, we investigated whether HA conjugation enhances the intrinsic antiproliferative activity of EGCG and enables synergistic growth inhibition when combined with cafestol in human renal cancer cells. The HA–EGCG conjugate was prepared using HA with a molecular weight of 90 kDa, which was selected to balance aqueous solubility, conjugate formation, and cellular interaction while minimizing the intrinsic biological effects associated with HA itself.

**Figure 1:**
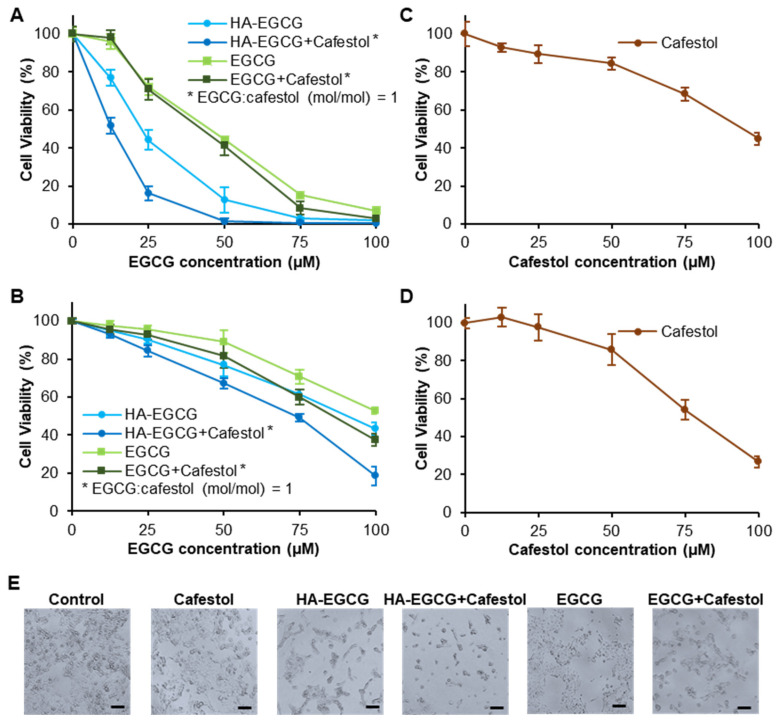
Anti-proliferative effects of free EGCG, HA–EGCG conjugate, cafestol and their combinations in human renal cancer cells. Cell viability of (**A**) ACHN and (**B**) A498 renal cancer cells treated with free EGCG or the HA–EGCG conjugate in the presence or absence of cafestol at an EGCG:cafestol molar ratio of 1:1 as a function of concentration for 48 h. Cell viability of (**C**) ACHN and (**D**) A498 cells treated with cafestol alone for 48 h. Data are presented as mean ± SD (*n* = 10–20). (**E**) Representative phase-contrast images of ACHN cells treated with cafestol, free EGCG, the HA–EGCG conjugate, and their combinations at 50 μM (EGCG:cafestol molar ratio = 1:1). Scale bar = 100 μm.

ACHN renal cancer cells were treated with increasing concentrations of free EGCG and the HA–EGCG conjugate. Free EGCG induced a dose-dependent reduction in cell viability, from 96% at 12.5 μM to 7.1% at 100 μM (Figure 1A), consistent with previous reports describing the antiproliferative effects of EGCG in renal cancer cells [23,24]. Notably, the HA–EGCG conjugate exhibited significantly lower cell viability than free EGCG across all tested concentrations, when normalized to the molar concentration of the EGCG moiety in the conjugate, with viability decreasing from 77% at 12.5 μM to 1.8% at 100 μM. Cafestol alone also suppressed ACHN cell proliferation in a dose-dependent manner (Figure 1C), consistent with earlier findings in renal cancer cells [25]. Importantly, co-treatment with cafestol and the HA–EGCG conjugate further reduced cell viability compared with treatment with the HA–EGCG conjugate alone. On the other hand, the combination of cafestol with free EGCG did not significantly improve growth inhibition relative to free EGCG alone.

To further evaluate whether these effects were reproducible across renal cancer models, antiproliferative activity was additionally assessed in A498 cells (Figure 1B,D). Similar to ACHN cells, treatment with the HA–EGCG conjugate resulted in greater growth inhibition than free EGCG alone, indicating that HA conjugation enhances the antiproliferative activity of EGCG in multiple renal cancer cell lines. Cafestol also reduced A498 cell viability in a dose-dependent manner. Co-treatment with cafestol and the HA–EGCG conjugate further enhanced growth inhibition compared with HA–EGCG conjugate alone, although the magnitude of enhancement was less pronounced than that observed in ACHN cells. These findings indicate that HA conjugation consistently improves the antiproliferative activity of EGCG in renal cancer cells, while the extent of the enhanced response to combination treatment may vary depending on the cellular context.

Representative phase-contrast images of ACHN cells treated with cafestol, EGCG, HA–EGCG conjugate, and their combinations at 50 μM (EGCG:cafestol (mol/mol) = 1) are shown in Figure 1E. The combination of cafestol and the HA–EGCG conjugate induced more pronounced morphological alterations and reduced cell density compared with the corresponding single treatments, consistent with the observed antiproliferative effects.

Unmodified HA alone showed negligible antiproliferative activity over the tested concentration range (Figure S1A), and its combination with cafestol produced effects comparable to cafestol alone (Figure S1B), indicating that the enhanced growth inhibition observed for the HA–EGCG conjugate can be attributed to the EGCG moiety rather than the HA backbone itself.

To further evaluate the influence of the combination ratio on antiproliferative activity, ACHN cells were treated with combinations of cafestol and the HA–EGCG conjugate at various EGCG:cafestol molar ratios (4:1, 2:1, 1:1, and 0.5:1) (Figure S2A). All tested combinations exhibited greater growth inhibition than the HA–EGCG conjugate alone, although the magnitude of enhancement varied depending on the combination ratio. CI analysis further demonstrated that synergistic interactions were most pronounced at the 1:1 molar ratio, whereas other ratios exhibited weaker or less consistent synergistic effects (Figure S2B). These findings suggest that the antiproliferative efficacy of the combination is influenced not only by HA conjugation but also by the relative ratio between the HA–EGCG conjugate and cafestol. Such ratio-dependent behavior is consistent with the general principles of combination therapy, in which the extent of synergistic interaction may depend on the relative contribution of each agent to the overall biological response.

The significantly enhanced antiproliferative activity of the HA–EGCG conjugate compared with free EGCG suggests that HA conjugation plays a critical role in improving the functional efficacy of EGCG. HA conjugation may enhance the cellular effects of EGCG through multiple mechanisms, potentially including improved molecular stability and sustained cellular exposure. In addition, HA is known to interact with receptors such as CD44, which are frequently expressed in renal cancer cells, and receptor-mediated interactions may further contribute to altered cellular handling of the conjugate. Collectively, these factors likely underlie the superior growth inhibition observed with the HA–EGCG conjugate and provide a mechanistic basis for the synergistic effects examined in the following sections.

### 2.2. Synergistic Inhibition of Renal Cancer Cell Proliferation

**Figure 2:**
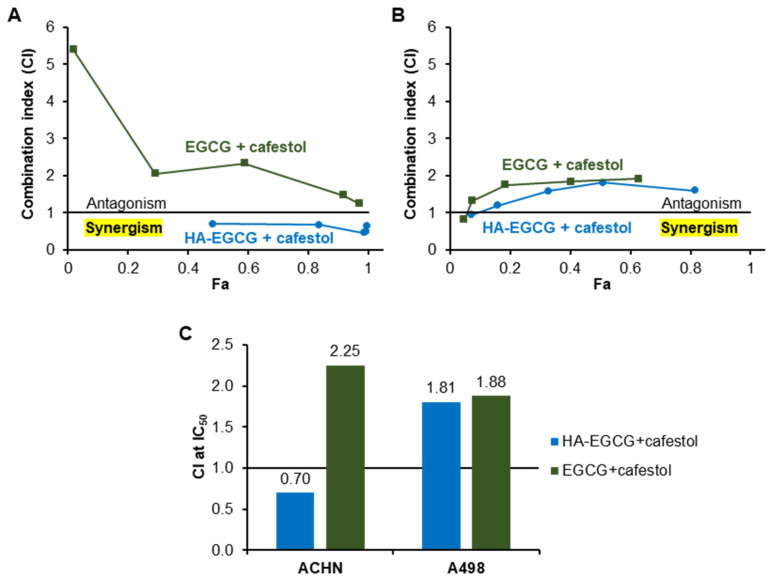
Combination index (CI) analysis of cafestol in combination with free EGCG or HA–EGCG conjugate in human renal cancer cells. CI values were calculated using the Chou–Talalay method for combinations of cafestol with free EGCG or the HA–EGCG conjugate at an EGCG:cafestol molar ratio of 1:1 in (**A**) ACHN and (**B**) A498 cells and plotted against the fraction affected (Fa), defined as x/100 at x% inhibition of cell proliferation. (**C**) Comparison of CI values at IC_50_ (Fa = 0.5) of combinations containing cafestol with free EGCG or the HA–EGCG conjugate in ACHN and A498 cells.

To quantitatively evaluate the combination effects of cafestol with free EGCG or HA–EGCG conjugate, the combination index (CI) was calculated using the Chou–Talalay method [26,27], based on their inhibitory effects on cell proliferation at various concentrations. In ACHN cells, the combination of cafestol and the HA–EGCG conjugate yielded CI values consistently below 1 (0.46–0.70) across all tested fraction affected (Fa) ranges (~0.5–1.0, corresponding to 50–100% cell proliferation inhibition), indicating synergistic antiproliferative activity (Figure 2A). In contrast, the combination of cafestol with free EGCG exhibited antagonistic or non-synergistic interactions, with CI values exceeding 2 (2.1–5.4) at low Fa (0.01–0.6) and remaining above 1 (1.2–1.5) even at high Fa values (>0.9), indicating an absence of synergistic effects. Consistent with the Fa–CI analysis, comparison of CI values at IC_50_ further demonstrated the distinct combination behaviors of the two treatments in ACHN cells (Figure 2C). The combination of cafestol and the HA–EGCG conjugate exhibited a substantially lower CI value than the combination of cafestol and free EGCG, supporting the emergence of synergistic interactions specifically following HA conjugation. Correspondingly, the combination of cafestol and the HA–EGCG conjugate reduced the half maximal inhibitory concentration (IC_50_) in ACHN cells by 1.7-fold, from 22.8 μM for the HA–EGCG conjugate alone to 13.1 μM for the combination treatment, whereas the combination of cafestol with free EGCG resulted in only a limited reduction in IC_50_ (Table S1).

In A498 cells, both combinations exhibited CI values close to or above 1 across the tested Fa range, indicating largely additive or non-synergistic interactions (Figure 2B). Similarly, CI values at IC_50_ in A498 cells remained substantially higher than those observed in ACHN cells and showed only limited differences between the two combination treatments (Figure 2C). Correspondingly, only modest reductions in IC_50_ values were observed following combination treatment in A498 cells (Table S1). These findings suggest that the synergistic interaction between cafestol and the HA–EGCG conjugate is cell line-dependent and more pronounced in ACHN cells, highlighting the influence of biological heterogeneity among renal cancer models on combination treatment responses.

The emergence of synergism specifically in HA–EGCG conjugate-treated ACHN cells suggests that HA conjugation plays an important role in enabling cooperative antiproliferative activity with cafestol. HA conjugation may alter the functional activity of EGCG through mechanisms such as improved resistance to autoxidation, sustained cellular exposure, and altered cellular interactions, thereby creating conditions that facilitate complementary anticancer activity by cafestol. In contrast, the limited effective cellular activity of free EGCG may restrict cooperative interactions, preventing the establishment of synergistic antiproliferative effects.

### 2.3. Selective Cytotoxicity Toward Renal Cancer Cells

**Figure 3:**
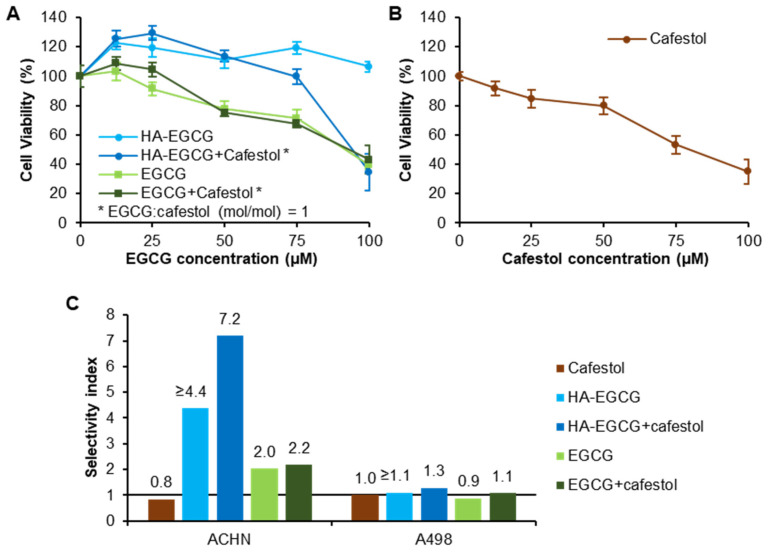
Selective cytotoxicity of free EGCG, HA–EGCG conjugate, cafestol and their combinations toward renal cancer cells. (**A**) Cell viability of normal renal epithelial RPTECs treated with free EGCG or the HA–EGCG conjugate in the presence or absence of cafestol at an EGCG:cafestol molar ratio of 1:1, as a function of concentration for 48 h. (**B**) Cell viability of RPTECs treated with cafestol alone for 48 h. (**C**) Selectivity index (SI) values of different treatments in ACHN and A498 cells relative to RPTECs. SI was calculated as IC_50_ in RPTEC/IC_50_ in cancer cells using the IC_50_ values summarized in Table S1. For the HA–EGCG conjugate in ACHN cells, the IC_50_ value in RPTECs was not reached within the tested concentration range (>100 μM), and the SI value is therefore presented as a minimum estimate. Data are presented as mean ± SD (*n* = 5–10).

To evaluate whether the enhanced antiproliferative activity of the HA–EGCG conjugate and its combination with cafestol was accompanied by selective cytotoxicity toward cancer cells, cytotoxic effects were assessed in normal renal proximal tubule epithelial cells (RPTECs). The HA–EGCG conjugate showed limited cytotoxicity toward RPTECs over the tested concentration range. The combination of cafestol with the HA–EGCG conjugate maintained substantially lower cytotoxicity toward RPTECs than cafestol alone (Figure 3A), despite producing greater antiproliferative effects in renal cancer cells (Figure 2). Free EGCG and its combination with cafestol produced moderate cytotoxic effects in RPTECs. Cafestol alone also reduced RPTEC viability in a dose-dependent manner (Figure 3B), with cytotoxicity comparable to that observed in renal cancer cells, indicating limited cancer cell selectivity for cafestol as a single agent.

To quantitatively compare cancer cell selectivity, selectivity index (SI) values were calculated as the ratio of IC_50_ values in RPTECs to those in renal cancer cells using the IC_50_ values summarized in Table S1. In ACHN cells, the HA–EGCG conjugate exhibited a higher SI value than free EGCG, and the combination of cafestol with the HA–EGCG conjugate produced the highest SI among all tested treatments (Figure 3C). Notably, although the combination treatment exhibited greater cytotoxicity toward RPTECs than the HA–EGCG conjugate alone, it retained substantially greater selectivity toward ACHN cells than cafestol alone. Because the IC_50_ value of the HA–EGCG conjugate in RPTECs was not reached within the tested concentration range (>100 μM), the corresponding SI value is presented as a minimum estimate. In contrast, SI values for A498 cells remained close to unity across all treatments, indicating limited selectivity toward A498 cells compared with RPTECs (Figure 3C).

These findings indicate that HA conjugation improves not only the antiproliferative activity of EGCG but also its selectivity toward renal cancer cells, particularly in ACHN cells. Together with the CI analysis, these results suggest that the beneficial effects of the HA–EGCG and cafestol combination, including synergy and selectivity, are cell line-dependent and most pronounced in ACHN cells.

### 2.4. Induction of Apoptosis in Renal Cancer Cells

**Figure 4:**
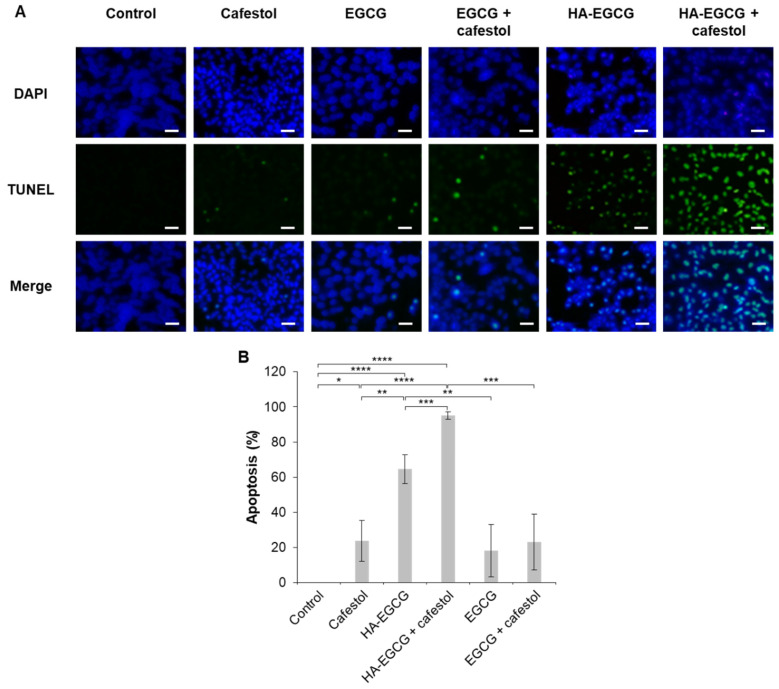
Induction of apoptosis in human renal cancer cells. (**A**) Representative fluorescence images of apoptotic cells (green, TUNEL staining) and nuclei (blue, DAPI staining) in ACHN cells treated with free EGCG, the HA–EGCG conjugate, cafestol, and their combinations at 12.5 μM (EGCG:cafestol molar ratio = 1:1) for 48 h. Scale bar = 100 μm. (**B**) Quantitative analysis of apoptotic cells. Data are presented as mean ± SD (*n* = 3). Statistically significant differences between groups are indicated by asterisks (* *p* < 0.05; ** *p* < 0.01; *** *p* < 0.005; **** *p* < 0.001).

Based on the pronounced synergistic and selective antiproliferative effects observed in ACHN cells, subsequent mechanistic studies were conducted using the ACHN model. To determine whether the enhanced antiproliferative effects were associated with apoptosis induction, DNA fragmentation was evaluated using a terminal deoxynucleotidyl transferase dUTP nick end labeling (TUNEL) assay. Control cells exhibited minimal TUNEL-positive staining, indicating low basal levels of apoptosis (Figure 4A,B). Treatment with free EGCG or cafestol alone resulted in a modest increase in apoptotic cells (18.1 and 23.7%, respectively) at the same concentration of 12.5 μM, consistent with their reported pro-apoptotic activity [25,28,29].

In contrast, treatment with the HA–EGCG conjugate induced a substantially higher proportion of TUNEL-positive nuclei (64.5%) compared with free EGCG, indicating that HA conjugation significantly enhanced the pro-apoptotic activity of EGCG. Notably, the combination of cafestol and the HA–EGCG conjugate produced the most pronounced apoptotic response (95.0%), exceeding the effects observed with either agent alone or with the combination of cafestol and free EGCG. These results suggest that HA conjugation enhances the functional pro-apoptotic activity of EGCG, likely by improving its resistance to autoxidation and sustaining effective cellular exposure, thereby sensitizing ACHN cells to the cooperative apoptotic effects of cafestol.

### 2.5. Downregulation of Anti-Apoptotic Factors

Cafestol and EGCG have been reported to promote apoptosis in cancer cells through modulation of survival-associated processes, including suppression of anti-apoptotic Bcl-2 family members [25,29,30,31]. Based on these reported activities, the expression of representative anti-apoptotic factors was examined to further elucidate the molecular basis of apoptosis induction in ACHN cells.

**Figure 5:**
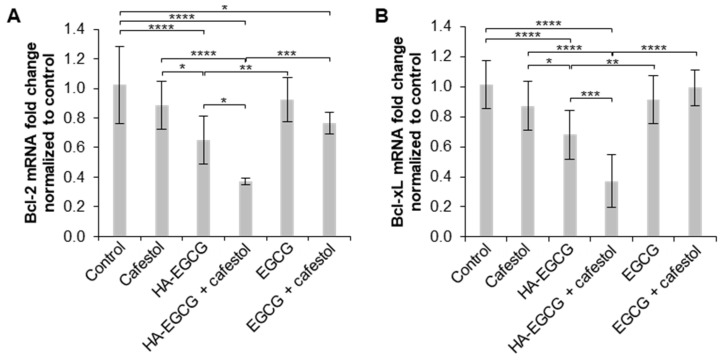
Downregulation of anti-apoptotic factors in human renal cancer cells. mRNA expression of anti-apoptotic factors (**A**) Bcl-2 and (**B**) Bcl-xL in ACHN cells treated with free EGCG, the HA–EGCG conjugate, cafestol, and their combinations (EGCG:cafestol molar ratio = 1:1). mRNA expression levels were assessed after 48 h of treatment. Data are presented as mean ± SD (*n* = 3). Statistically significant differences between groups are indicated by asterisks (* *p* < 0.05; ** *p* < 0.01; *** *p* < 0.005; **** *p* < 0.001).

Quantitative real-time PCR analysis showed that treatment with free EGCG or cafestol alone (12.5 μM) resulted in only modest reductions in Bcl-2 and Bcl-xL mRNA expression compared with untreated controls (Figure 5A,B). In contrast, treatment with the HA–EGCG conjugate led to significantly greater downregulation of both transcripts. Importantly, the combination of cafestol and the HA–EGCG conjugate produced the most pronounced suppression of Bcl-2 and Bcl-xL mRNA expression. These molecular changes are consistent with the enhanced apoptotic response observed in ACHN cells treated with the HA–EGCG conjugate, particularly in combination with cafestol.

Western blot analysis of Bcl-2 and Bcl-xL was additionally performed. However, although modest changes in band intensity were observed, the results did not consistently demonstrate statistically significant differences across independent experiments. Therefore, to avoid overinterpretation of marginal or insufficiently reproducible protein-level changes, this study places primary emphasis on the quantitative PCR results for Bcl-2 and Bcl-xL.

### 2.6. Downregulation of EMT-Related Factors

Beyond apoptosis regulation, cafestol and EGCG have been reported to influence cellular processes associated with epithelial–mesenchymal transition (EMT), which plays a critical role in renal cancer progression, cellular plasticity, therapeutic resistance, and cell migration [25,32]. EMT-associated transcription factors such as SNAIL serve as key regulators of these processes, promoting mesenchymal traits and enhanced migratory potential, and therefore represent relevant molecular targets for evaluation [8,9].

**Figure 6:**
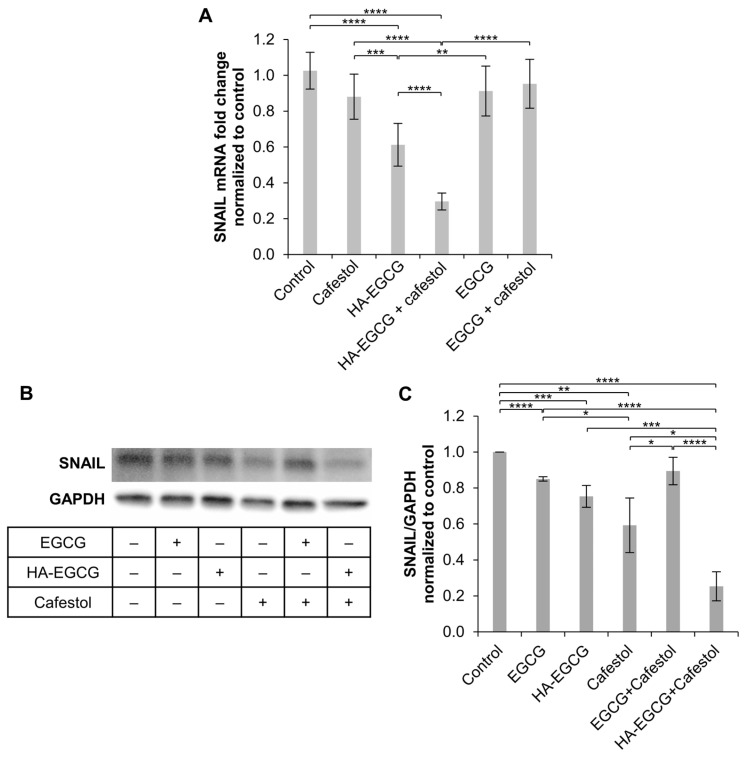
Downregulation of EMT-related factors in human renal cancer cells. (**A**) mRNA expression and (**B**) protein levels and (**C**) corresponding quantitative analysis of the EMT-associated transcription factor SNAIL in ACHN cells treated with free EGCG, the HA–EGCG conjugate, cafestol, and their combinations (EGCG:cafestol molar ratio = 1:1). mRNA expression and protein levels were assessed after 48 h and 24 h of treatment, respectively. Data are presented as mean ± SD (*n* = 3). Statistically significant differences between groups are indicated by asterisks (* *p* < 0.05; ** *p* < 0.01; *** *p* < 0.005; **** *p* < 0.001).

In the present study, treatment with free EGCG or cafestol alone (12.5 μM) resulted in only minor changes in SNAIL mRNA expression (Figure 6A). In contrast, the HA–EGCG conjugate markedly enhanced the suppression of SNAIL expression. Notably, the combination of cafestol and the HA–EGCG conjugate produced the most pronounced downregulation of SNAIL at both the transcriptional and protein levels, as confirmed by Western blot analysis and corresponding band quantification (Figure 6B,C), whereas the physical mixture of cafestol and free EGCG failed to achieve comparable effects. Given the established role of SNAIL in driving EMT-associated cellular plasticity and migratory behavior, the observed suppression of SNAIL by the combination of cafestol and the HA–EGCG conjugate suggests coordinated inhibition of EMT-related transcriptional programs.

Collectively, these results demonstrate that conjugation of EGCG to HA is a key enabling factor for its synergistic interaction with cafestol in renal cancer cells, particularly ACHN cells, leading to markedly enhanced anticancer activity. HA conjugation significantly improved the antiproliferative efficacy of EGCG and enabled strong cooperative effects with cafestol, allowing effective growth inhibition at reduced concentrations compared with either agent alone. This concentration-sparing effect is therapeutically relevant, as it may help mitigate dose-dependent toxicities associated with high levels of EGCG or cafestol while preserving or enhancing anticancer potency. The enhanced functional activity of the HA–EGCG conjugate may arise from multiple factors, including improved resistance to autoxidation, prolonged effective cellular exposure, and altered cellular interactions following HA conjugation. Interactions between HA and receptors such as CD44 may also contribute to the enhanced cellular effects of the conjugate, although the involvement of CD44 was not specifically evaluated in the present study.

**Figure 7:**
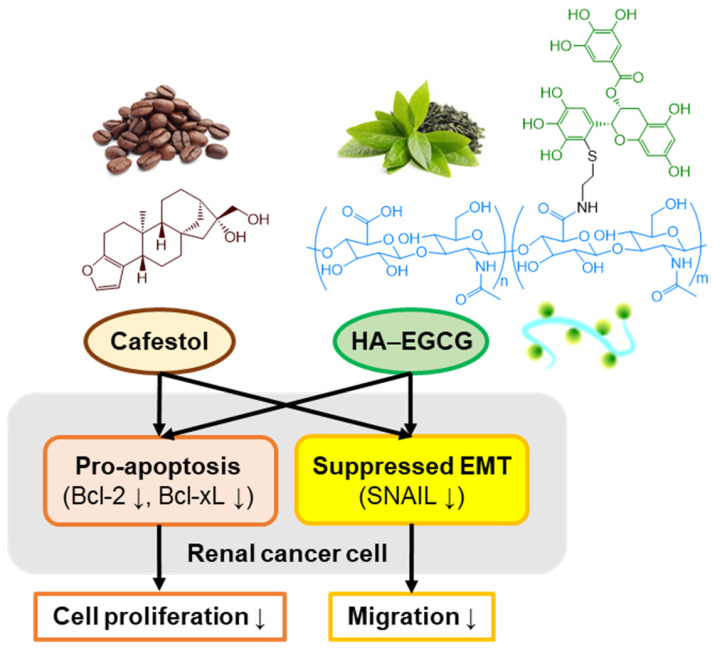
Schematic illustration of the proposed synergistic anticancer mechanisms of cafestol and the HA–EGCG conjugate. Cafestol and the HA–EGCG conjugate cooperatively suppress renal cancer cell proliferation and EMT-associated transcriptional programs through coordinated induction of apoptosis and inhibition of EMT-related signaling pathways (orange- and yellow-colored molecules, respectively).

Beyond proliferation inhibition, the synergistic interaction between cafestol and the HA–EGCG conjugate was accompanied by coordinated modulation of multiple cancer-associated processes, including enhanced apoptosis, downregulation of anti-apoptotic factors, and suppression of the EMT-associated transcription factor SNAIL, as schematically summarized in Figure 7. Given the central roles of apoptosis resistance and EMT in renal cancer progression, cellular plasticity, and therapeutic resistance, such multi-mechanistic engagement may provide a more robust strategy for limiting adaptive resistance and improving treatment durability.

Although enhanced antiproliferative activity was observed in both ACHN and A498 cells, synergistic and selective effects were more pronounced in ACHN cells, suggesting potential cell line-dependent responses. Because the present study was conducted using two renal cancer cell lines with distinct biological characteristics, further evaluation in additional renal cancer models and subtypes may help better reflect the molecular heterogeneity of renal cancer. In addition, the current mechanistic analysis focused primarily on apoptosis- and EMT-related regulation, while evaluation of additional molecular pathways and markers such as caspases, E-cadherin, and N-cadherin may provide further insight into the biological effects of the HA–EGCG conjugate and its combination with cafestol.

Importantly, both EGCG and cafestol are naturally occurring bioactive compounds with established dietary exposure, providing contextual relevance for their biological activity. EGCG is the predominant catechin in green tea, with reported contents of approximately 50–300 mg per cup, depending on tea variety and brewing conditions [33,34]. However, pharmacokinetic studies indicate that consumption of green tea alone typically results in plasma EGCG concentrations in the submicromolar range (generally <0.5 μM), reflecting limited systemic bioavailability due to chemical instability, extensive first-pass metabolism, and rapid clearance [35]. Similarly, cafestol is present in unfiltered coffee preparations, such as French press or espresso, at levels of approximately 1–4 mg per cup, with estimated intestinal absorption of ~70%; nevertheless, circulating cafestol concentrations following usual coffee consumption are also expected to remain within the low micromolar or submicromolar range [36,37]. These observations suggest that the concentration ranges at which EGCG and cafestol exert anticancer effects are unlikely to be achieved through habitual green tea or coffee consumption alone [35,36].

In this context, the present findings demonstrate that conjugation of EGCG to HA markedly enhances its functional anticancer efficacy and enables synergistic cooperation with cafestol at reduced effective concentrations, potentially helping bridge the gap between dietary exposure and biologically relevant intracellular activity. Such concentration-sparing effects highlight the potential translational relevance of this combination strategy and support further evaluation in additional renal cancer cell models and appropriate in vivo systems to evaluate pharmacokinetics, biodistribution, metabolic behavior, therapeutic efficacy, and safety.

## 3. Materials and Methods

### 3.1. Materials

Cafestol was purchased from Santa Cruz Biotechnology (Dallas, TX, USA). Epigallocatechin-3-O-gallate (EGCG, purity > 95%) was obtained from Euro Chem-Pharma Sdn Bhd (Shah Alam, Malaysia). Hyaluronic acid (HA, 90 kDa) was obtained from JNC Corporation (Tokyo, Japan). The following TaqMan PCR primers were used for mRNA analysis: Bcl-2 (Hs00608023_m1), Bcl-xL (Hs99999146_m1), SNAIL (Hs00195591_m1), and GAPDH (Hs02758991_g1) from Thermo Fisher Scientific (Waltham, MA, USA). The following antibodies were used during Western blot analyses: mouse anti-Bcl-2 (15071S), rabbit anti-Bcl-xL (2764S), rabbit anti-SNAIL (3879S), and horseradish peroxidase (HRP)-conjugated anti-rabbit IgG (7074V) antibodies from Cell Signaling Technology (Danvers, MA, USA); mouse anti-GAPDH (60004-1-Ig) and HRP-conjugated anti-mouse IgG (H+L) (SA00001-1) antibodies from Proteintech (Rosemont, IL, USA). All other chemicals were of analytical grade.

### 3.2. Synthesis of the HA–EGCG Conjugate

Hyaluronic acid (HA, 90 kDa) was used for conjugation. The HA–EGCG was synthesized using a previously reported thiol-mediated conjugation method [38]. Briefly, HA was reacted with cystamine for 24 h to obtain thiolated HA (HA–SH), followed by purification via dialysis against water. HA–SH was then redissolved and reacted with EGCG in an aqueous solution under basic pH conditions for 5 h. The resulting HA–EGCG conjugate was purified by dialysis and lyophilized. The degree of substitution (DS), the number of -EGCG substituents per 100 repeating units of HA, was determined by measuring the absorbance of EGCG at 274 nm using a U-2810 spectrophotometer (Jasco V770 spectrophotometer, Jasco Corporation, Tokyo, Japan). The DS of the HA–EGCG conjugate that was used in this study was 6.4. Physicochemical characterization of HA–EGCG, including dynamic light scattering (DLS) analysis and autoxidation stability in PBS, is provided in Table S2 and Figure S3.

### 3.3. Cell Culture

Human renal cancer ACHN and A498 cells were obtained from ATCC (Manassas, VA, USA) and cultured in Dulbecco’s Modified Eagle’s Medium (DMEM) supplemented with 10% fetal bovine serum (FBS) and 1% penicillin–streptomycin. Primary human renal proximal tubule epithelial cells (RPTECs) were obtained from ATCC (Manassas, VA, USA) and cultured in renal epithelial cell basal medium (ATCC^®^ PCS-400–030™) supplemented with a renal epithelial cell growth kit (ATCC^®^ PCS-400–040™).

### 3.4. Evaluation of Antiproliferative Activity and Cytotoxicity

Cells were seeded (5 × 10^3^ cells/well) in quadruplicate in 96-well microplates and allowed to adhere for 24 h. The culture media were then replaced with media containing the following samples: cafestol, EGCG, HA–EGCG conjugate, the physical mixture of cafestol and EGCG or HA–EGCG conjugate (12.5–100 µM of cafestol or EGCG moiety), HA, or the physical mixture of cafestol and HA. The concentrations of HA correspond to the amount of HA in the HA–EGCG conjugate. The cells were incubated at 37 °C in 5% CO_2_. After 48 h, cell viability was measured by using AlamarBlue reagent (Life Technologies, Carlsbad, CA, USA) according to the manufacturer’s protocol. Briefly, the medium was replaced with a medium containing 10% AlamarBlue reagent. After incubation for 4 h at 37 °C, cell viability was determined by monitoring the fluorescence intensity (λex = 549 nm and λem = 587 nm) using a microplate reader (Tecan Group Ltd., Männedorf, Switzerland). Results were expressed as a percentage of viable cells relative to untreated cells. Representative cell images were acquired using an Olympus CKX53 inverted microscope (Tokyo, Japan).

### 3.5. Synergism Quantitation

The quantitative analysis of the combined effects of cafestol and EGCG or the HA–EGCG conjugate on the cell proliferation inhibitory effect was performed using the combination index (CI) based on the Chou and Talalay method, which has been widely used for quantifying in vitro and in vivo drug combinations [26,27], where CI < 1 indicates synergism, CI = 1 indicates additive effects, and CI > 1 indicates antagonism. The CI values of the combinations were calculated with the following equation using the CompuSyn software (Version 1.0) [39].

$$\mathrm{CI}=\frac{{\left(D\right)}_{\mathrm{cafestol}}}{{\left(D_{x}\right)}_{\mathrm{cafestol}}}+\frac{{\left(D\right)}_{\mathrm{EGCG}\;\mathrm{or}\;\mathrm{HA}–\mathrm{EGCG}}}{{\left(D_{x}\right)}_{\mathrm{EGCG}\;\mathrm{or}\;\mathrm{HA}–\mathrm{EGCG}}}$$

where (D)_cafestol_ and (D)_EGCG or HA–EGCG_ are the doses of cafestol and EGCG or HA–EGCG conjugate in the combination to achieve x% effects, and (D_x_)_cafestol_ and (D_x_)_EGCG or HA–EGCG_ are the doses of cafestol alone and EGCG alone or the HA–EGCG conjugate alone to achieve the same effect.

### 3.6. Evaluation of Selectivity Index

Selective cytotoxicity toward renal cancer cells was evaluated by comparing the antiproliferative effects of treatments in renal cancer cells (ACHN and A498) and normal renal proximal tubule epithelial cells (RPTEC). Half maximal inhibitory concentration (IC_50_) values were calculated from dose–response curves using nonlinear regression analysis. The selectivity index (SI) was calculated according to the following equation.

$$\mathrm{SI}=\frac{{\mathrm{IC}}_{50,\;\mathrm{RPTEC}}}{{\mathrm{IC}}_{50,\;\mathrm{cancer}}}$$

where IC_50, RPTEC_ and IC_50, cancer_ represent the IC_50_ values obtained in RPTECs and renal cancer cells, respectively. Higher SI values indicate greater selectivity toward cancer cells relative to normal renal epithelial cells. For treatments in which the IC_50_ value in RPTECs was not reached within the tested concentration range, the SI value was presented as a minimum estimate based on the maximum tested concentration.

### 3.7. Apoptosis Assay

Apoptosis was detected using a terminal deoxynucleotidyl transferase (TdT) dUTP nick end labeling (TUNEL) assay with the In Situ Cell Death Detection Kit, Fluorescein (Sigma-Aldrich, St. Louis, MO, USA), as per the manufacturer’s instructions. Briefly, ACHN cells (1.5 × 10^4^ cells in an 8-well chamber slide) were exposed to cafestol, EGCG, the HA–EGCG conjugate, or the physical mixture of cafestol and EGCG or the HA–EGCG conjugate (12.5 µM of cafestol or EGCG moiety) for 48 h. The cells were then fixed with 4% (*w*/*v*) paraformaldehyde at 25 °C for 1 h, washed with PBS, and permeabilized with 0.1% Triton X-100 in 0.1% sodium citrate at 4 °C for 2 min. After two washes with PBS, the cells were incubated with TUNEL reaction mixture containing the TdT and nucleotide mixture at 37 °C for 1 h in a humidified atmosphere in the dark. After three washes with PBS, an aqueous mounting medium with DAPI, Fluoroshield (Abcam, Cambridge, UK), was added to the cells. Fluorescence microscopy images were acquired using an Olympus CKX53 inverted microscope (Tokyo, Japan).

### 3.8. Analysis of mRNA Expression

ACHN cells were seeded in 6 cm dishes (3 × 10^5^ cells/dish, *n* = 3) and allowed to adhere for 1 day. The culture media were then replaced with media containing the following samples: cafestol, EGCG, the HA–EGCG conjugate, or the physical mixture of cafestol and EGCG or the HA–EGCG conjugate (12.5 µM of cafestol or EGCG moiety). After 48 h, the cells were collected for evaluation of the mRNA expression level. Total RNA was extracted from the samples with the RNAiso Plus reagent (Takara Bio Inc., Kusatsu, Japan) according to the manufacturer’s protocol. The total RNA yield, purity and concentration were determined using a DS-11 Spectrophotometer (DeNovix, Wilmington, DE, USA). For the preparation of cDNA, total RNA was incubated with the ReverTra AceTM qPCR RT kit reaction mixture (Toyobo, Tokyo, Japan), containing a reverse transcription buffer, a primer mixture and an enzyme mixture. Real-time quantitative polymerase chain reaction (PCR) was conducted using the Mx3000P qPCR system (Agilent Stratagene, La Jolla, CA, USA). The reaction mixtures contained 10 μL of THUNDERBIRDTM Probe qPCR Mix, 0.04 μL of 50× ROX reference dye (Toyobo, Tokyo, Japan), 1 μL of each TaqMan PCR primer, 2 μL of cDNA, and 6.96 μL of diethyl-pyrocarbonate (DEPC)-treated water. The thermal profile used for PCR was 95 °C for 10 min, followed by 40 cycles of 95 °C for 15 s and 60 °C for 60 s. The average threshold cycle (Ct) values of triplicate measurements were used in all subsequent calculations using the delta-delta Ct method, and the results are presented as the fold change in gene expression normalized to the endogenous reference gene glyceraldehyde phosphate dehydrogenase (GAPDH).

### 3.9. Western Blot Analysis

ACHN cells were seeded in 6-well plates (1.5 × 10^5^ cells/well, *n* = 3) and allowed to adhere for 1 day. The culture media were then replaced with media containing the following samples: cafestol, EGCG, HA–EGCG conjugate, or the physical mixture of cafestol and EGCG or HA–EGCG conjugate at concentrations of 25 μM, based on the cafestol or EGCG moiety. After 24 h, cell lysates were prepared using a RIPA lysis buffer containing 1% protease inhibitor cocktail and phosphatase inhibitor cocktail (Atto, Japan). Soluble protein lysates (4 µg) were mixed with a lithium dodecyl sulfate sample buffer and sample reducing agent (Thermo Fisher Scientific, Waltham, MA, USA) and separated through sodium dodecyl sulfate polyacrylamide gel electrophoresis. The separated proteins were then transferred to PVDF membranes. The membranes were blocked with 1% gelatin and 0.05% Tween in Tris-buffered saline for 1 h at room temperature and then incubated overnight at 4 °C with a primary antibody according to the manufacturer’s instructions. After three washes, the membranes were incubated with an HRP-conjugated anti-rabbit or anti-mouse secondary antibody for 1 h at room temperature. Protein bands were detected using Clarity Max or Clarity Western ECL Substrate (Bio-Rad, Hercules, CA, USA).

### 3.10. Statistical Analysis

All data are presented as the mean ± standard deviation (SD). Statistical analysis was conducted using the OriginPro 2022 software (one-way analysis of variance (ANOVA) with Tukey’s post hoc test). A *p*-value less than 0.05 was considered statistically significant.

## 4. Conclusions

This study demonstrates that the conjugation of EGCG to HA markedly enhances its antiproliferative activity and enables a synergistic interaction with cafestol in human renal cancer cells. In particular, the HA–EGCG conjugate enhanced antiproliferative activity in both ACHN and A498 cells and exhibited pronounced synergistic effects in ACHN cells, effects that were not observed with free EGCG. In addition, the HA–EGCG conjugate and its combination with cafestol exhibited favorable selectivity toward renal cancer cells compared with non-malignant renal epithelial cells. This synergistic interaction was accompanied by the robust enhancement of apoptosis, the pronounced downregulation of anti-apoptotic factors, and the effective suppression of the EMT-associated transcription factor SNAIL.

The ability to achieve these effects at lower concentrations highlights the potential of this combination strategy to mitigate dose-related toxicity while maintaining anticancer activity. Moreover, the coordinated modulation of multiple cancer-associated processes suggests that this approach may provide a more effective strategy to limit tumor adaptability and therapeutic resistance. Collectively, these findings support HA-based conjugation of natural bioactive compounds as a promising multi-targeted platform for the development of improved therapeutic strategies against renal cancer.

## Data Availability

The original contributions presented in this study are included in the article/Supplementary Materials. Further inquiries can be directed to the corresponding authors.

## Supplementary Materials

The following supporting information can be downloaded at https://www.mdpi.com/article/10.3390/ijms27114929/s1.

## Abbreviations

The following abbreviations are used in this manuscript:

CI	Combination index
EGCG	Epigallocatechin gallate
EMT	Epithelial–mesenchymal transition
HA	Hyaluronic acid
IC_50_	Half maximal inhibitory concentration
ICIs	Immune checkpoint inhibitors
TdT	Terminal deoxynucleotidyl transferase
TKIs	Tyrosine kinase inhibitors
TUNEL	Terminal deoxynucleotidyl transferase (TdT) dUTP nick end labeling
