# Supplementary material for: Synergistic and Selective Antiproliferative Effects of Cafestol and a Hyaluronic Acid–Epigallocatechin Gallate Conjugate in Human Renal Cancer Cells
Source: Int J Mol Sci. 2026 May 29;27(11):4929. doi: 10.3390/ijms27114929 (PMC13256355; doi:10.3390/ijms27114929)
Supplement: Supplementary file 1 [file ijms-27-04929-s001.zip › ijms-4136519-supplementary.docx]

**Supplementary Information**

**Synergistic and Selective Antiproliferative Effects of Cafestol and a Hyaluronic Acid–Epigallocatechin Gallate Conjugate in Human Renal Cancer Cells**

**Nunnarpas Yongvongsoontorn ^1^, Yudo Sawa ^1^, Atsushi Yamashita ^1^, Joo Eun Chung ^1^, Kaoru Hiratsuka ^2^, Koji Izumi ^2^, Hiroaki Iwamoto ^2,^* and Motoichi Kurisawa ^1,^***

^1^ Graduate School of Advanced Science and Technology, Japan Advanced Institute of Science and
Technology, 1-1 Asahidai, Nomi 923-1292, Ishikawa, Japan; nyong@jaist.ac.jp (N.Y.);
s2410066@jaist.ac.jp (Y.S.); a-yama@jaist.ac.jp (A.Y.); chungje@jaist.ac.jp (J.E.C.)

^2^ Integrative Cancer Therapy and Urology, Graduate School of Medical Sciences, Kanazawa University,
13-1 Takara-machi, Kanazawa 920-8640, Ishikawa, Japan; hirakaoru@med.kanazawa-u.ac.jp (K.H.);
azui-zu2003@yahoo.co.jp (K.I.)

***** Correspondence: hiroaki017@yahoo.co.jp (H.I.); kurisawa@jaist.ac.jp (M.K.)


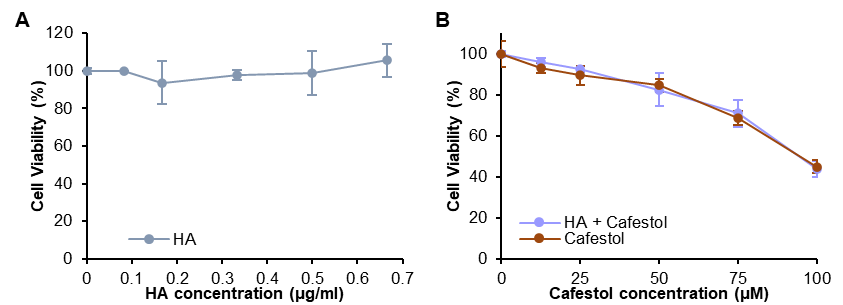


**Figure S1.** Anti-proliferative effects of HA alone and in combination with cafestol in human renal cancer cells. Cell viability of ACHN cells treated with (**A**) HA at concentrations equivalent to those used in the HA–EGCG conjugate, and (**B**) HA in combinations with cafestol, as a function of concentration for 48 h. Data are presented as mean ± sd (n = 5).


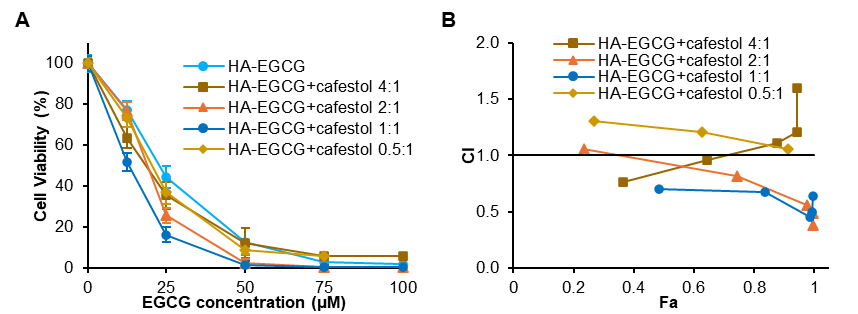


**Figure S2.** Ratio-dependent antiproliferative and combinational effects of HA–EGCG conjugate and cafestol in ACHN renal cancer cells. (**A**) Cell viability of ACHN cells treated with HA–EGCG conjugate in the presence or absence of cafestol at various EGCG:cafestol molar ratios (4:1, 2:1, 1:1, and 0.5:1) as a function of concentration for 48 h. (**B**) Combination index (CI) values for the corresponding combinations, calculated using the Chou–Talalay method and plotted against the fraction affected (Fa), defined as x/100 at x% inhibition of cell proliferation. Data are presented as mean ± SD (n = 5–20).


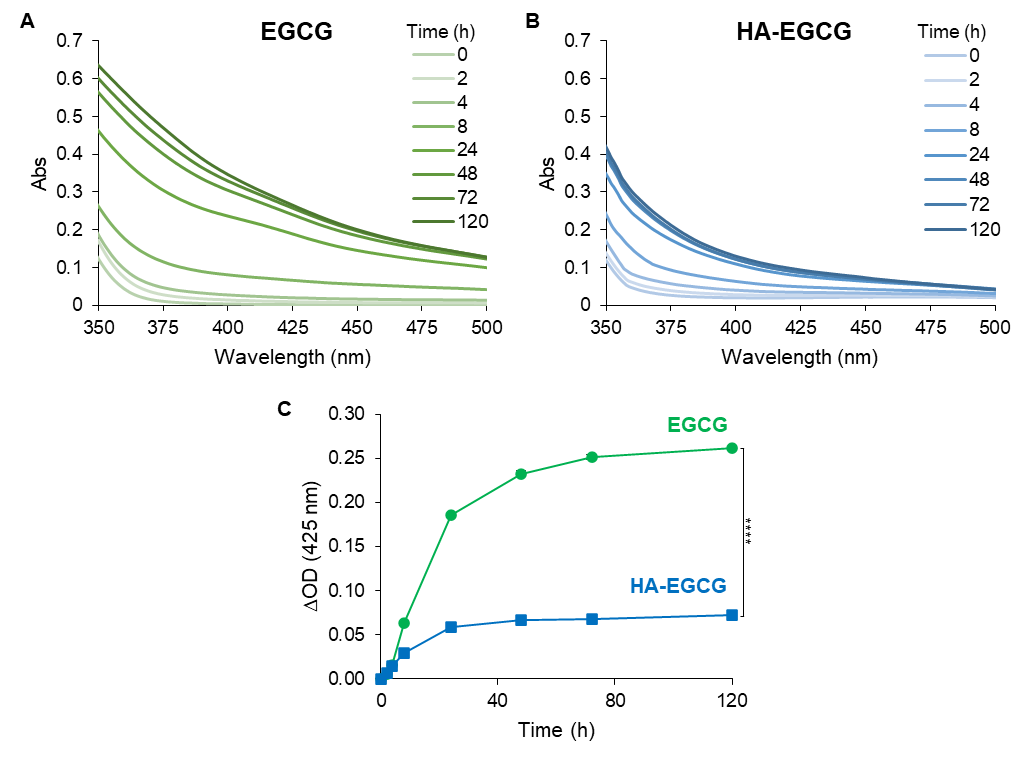


**Figure S3.** Improved autoxidation stability of HA–EGCG. Time-resolved UV–vis spectra of (A) EGCG and (B) HA–EGCG in PBS at 25°C. (C) The corresponding absorbance changes at 425 nm (ΔOD 425 nm) as a function of incubation time. Data are presented as mean ± SD (n = 3, *****p* < 0.001).

**Table S1.** IC₅₀ values (μM) of cafestol, EGCG, HA–EGCG conjugate, and their combinations at an EGCG:cafestol molar ratio of 1:1 in ACHN, A498, and RPTEC cells. For combination treatments, IC₅₀ values are expressed based on the concentration of EGCG or HA–EGCG equivalent EGCG concentration.

| **Treatment** | **ACHN** | **A498** | **RPTEC** |
| --- | --- | --- | --- |
| Cafestol | 94.7 | 78.7 | 79.3 |
| HA–EGCG | 22.8 | 90.7 | >100 |
| HA–EGCG + cafestol | 13.1 | 73.8 | 94.1 |
| EGCG | 45.0 | 103.9 | 91.7 |
| EGCG + cafestol | 42.6 | 86.0 | 93.0 |

**Table S2.** Physicochemical characterization of HA–EGCG conjugate dispersed in deionized water (0.2 mg/mL).

| Hydrodynamic diameter (nm) | 983.9 ± 73.1 |
| --- | --- |
| Polydispersity index | 0.500 ± 0.089 |
| Zeta potential (mV) | -59.2 ± 0.6 |
